# EpiMap: Fine-tuning integrative epigenomics maps to understand complex human regulatory genomic circuitry

**DOI:** 10.1038/s41392-021-00620-5

**Published:** 2021-05-08

**Authors:** Dave S. B. Hoon, Negin Rahimzadeh, Matias A. Bustos

**Affiliations:** Department of Translational Molecular Medicine, Division of Molecular Oncology, Saint John’s Cancer Institute at Providence Saint John’s Health Center, Santa Monica, CA USA

**Keywords:** Genome informatics, Epigenetics, Diseases

In a recent paper published in *Nature*, Boix et al.^1^ developed an integrated platform on epigenomic maps that represents a significant updated resource in human regulatory circuitry as related to specific organ tissues and cells, as well as specific disease traits. The EpiMap (for epigenome integration across multiple annotation projects) is a highly valuable compendium comprising 10,000 epigenomic maps across 833 biospecimens and 33 tissue categories. The distinct biospecimens expanding coverage to life development (embryonic to adult) and different sample types including complex organ tissues as well as primary cells, and established normal and tumor cell lines. The biospecimen analysis provides a comprehensive view of human genome circuitry making EpiMap a remarkable platform for epigenomic landscapes assessment to the investigator.

The imputed datasets used to build EpiMap are across 18 epigenomic marks well-annotated whose combination differentiates many classes of chromatin states, including enhancer, promoter, transcribed, repressed, and quiescent regions. The EpiMap consists of 2.1 million well-annotated high-resolution gene-regulatory regions as well as their activity patterns covering the biospecimens. The compendium also included enriched regulatory motifs, motif combinations, and putative upstream regulators that are responsible for their co-regulation, enriched gene functions, biological pathways of the gene-regulatory regions, and their tissue-specific target genes. The 2.1 million high-resolution active-enhancer regions annotated were obtained by intersecting five active-enhancer states with 3.6 million accessible DNA regions from 733 DNase-seq analyses. This provides an analysis of biospecimens with greater than twofold increase relative to the ENCODE phase III 2020 release.^2^ The study identified active enhancer marks that correlate with gene activity levels across 833 biospecimens, which helped to define 300 enhancer modules including 290 tissue-specific modules. These enhancer modules predicted tissue specific gene ontology enrichments.

By combining epigenomic–transcriptional correlation and genomic proximity 3.3 million tissue-specific enhancer–gene links were predicted. These links were sixfold more specific than enhancers and outperformed previous linking approaches, using both gene-set enrichment metrics and curated gold-standard datasets. Thus, offering a valuable and more accurate resource than previously reported.^2^

Genome-wide association studies (GWAS) have greatly contributed to understanding complex traits and disease-related phenotypes. For the last decade, GWAS landscape has evolved to map more than 100,000 genomic loci containing common single-nucleotide polymorphisms (SNP) that allow for interrogation of complex human diseases.^3^ However, as the authors point out the major limitation is that majority of these genetic associations remain devoid of any mechanistic *hypothesis* underlying their molecular and cellular functions. The main explanation is that >90% of these genetic variants lie on non-coding regions, and likely have unresolved gene-regulatory roles in cell circuitry.

In this study, the EpiMap was used as a source of large-scale mapping to inferred chromatin-states and high-resolution enhancer annotations that were applied to GWAS. To determine the potential role of epigenomic marks in controlling specific genomic loci and complex traits, the authors used the annotated 2.1 million enhancers and their tissue specificity. EpiMap overcomes many of GWAS limitations and provides a new human epigenome reference and a rich interactive supplementary website (http://compbio.mit.edu/epimap/). The compendium resource provides annotation of 30,000 genetic loci associated with 540 traits, predicting trait-relevant tissues, putative causal nucleotide variants in enriched tissue enhancers, and candidate tissue-specific target genes for each. This represents a tenfold increase compared to a previous report by Roadmap Epigenomic projects.

**Fig. 1:**
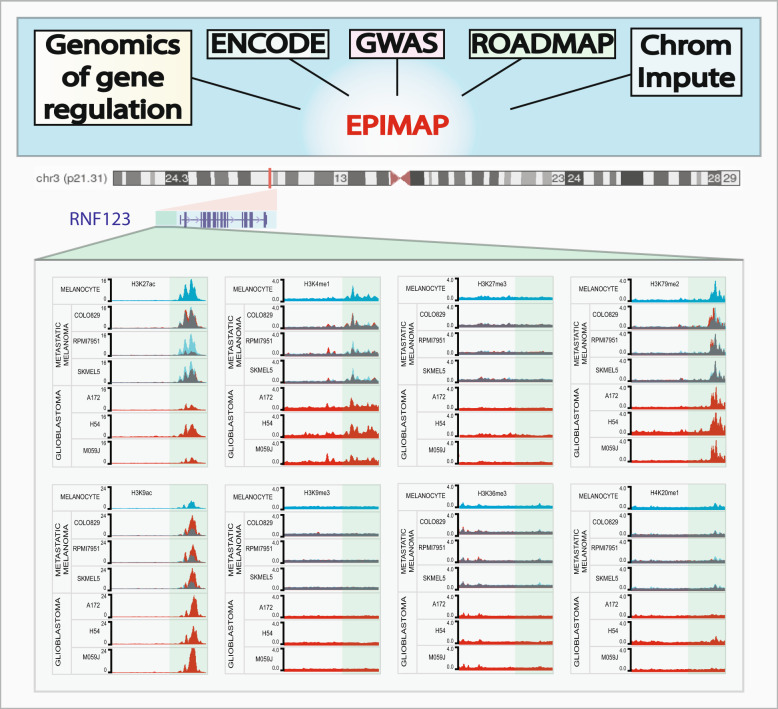
Histone marks on *RNF123* gene obtained from EpiMap. EpiMap is a compendium of epigenomes across multiple annotation projects: Genomics of gene regulation, ENCODE, ROADMAP, GWAS, and Chrom Impute. Presented are the histone modifications at the promoter region of *RNF123* gene (Chromosome 3, p21.31) in primary melanocytes (melanocytes), metastatic melanoma (COLO829, SK-MEL5, and RPMI7951) and glioblastoma cell lines (H54, M059J, and A172). All the tracks were downloaded from EpiMap and visualized on WASHU epigenome browser

To further demonstrate the utility of EpiMap, we focused on the *RNF123* gene that was previously reported by ours and Ciechanover’s group as downregulated in metastatic melanoma and glioblastoma tumors.^4,5^ RNF123, also known as KPC1, is an ubiquitin E3 ligase involved in ubiquitination and proteasomal processing of NF-ĸB1 p105 precursor to generate p50. RNF123 acts as a major tumor suppressor factor except in metastatic melanoma and glioblastoma. RNF123 downregulation results in a p50 decrease, which reduces the amount of the transcriptional inactive p50 dimers regulator, thereby promoting tumor growth and metastasis.^4,5^ The histone epigenomic marks were assessed to determine regulatory regions controlling *RNF123* expression in epidermal primary melanocytes and metastatic melanoma cell lines (COLO829, SK-MEL5, and RPMI7951) and glioblastoma cell lines (H54, M059J, and A172) (Fig. 1). Consistent with a decrease in RNF123 expression in metastatic tumor cell lines and tissues previously reported,^5^ we observed a significant reduction in H3K27Ac in metastatic melanoma and glioblastoma cell lines compared to primary melanocytes. However, and as we already demonstrated other specific epigenetic regulators such as microRNA, in this case miR-155-5p, also control *RNF123* mRNA expression at post-transcriptional levels.^5^ The two different tumor cells assessment provide evidence of the applicability of epigenomic regulatory marks to gene-regulation downloadable from the EpiMap.

The diverse classes of gene-regulatory annotations allows us to elucidate the molecular basis of complex traits by enabling: (1) detailed interactive exploration of functional and motif enrichments of enhancer modules, (2) motif-tissue networks and enrichments, (3) GWAS enrichments, (4) GWAS-enriched tissue enhancer SNP overlaps and target gene predictions, and (5) 30,000 disease locus visualizations with putative driver SNPs, enhancers, tissues, and tissue-specific target genes. These complex regulatory epigenomic maps also provide an interactive data analysis browser through the website, including biospecimens and track exploration, the creation of custom track hubs, modules, and motifs enrichments. The EpiMap compendium will provide an invaluable resource for the scientific community to interrogate non-coding functions and apply them to elevate investigations to higher levels, all of which will help to explain the epigenomic complexity of the human genome. The EpiMap also provides a framework for future data input to better understanding of regulatory cell circuitry.
